# Cross-Sectional Associations of Sport Participation, Academic Performance, and Psychological Well-Being Among Rural Romanian Adolescent Boys in the Context of Family Background

**DOI:** 10.3390/children13010135

**Published:** 2026-01-16

**Authors:** Filoména Dávid, Krisztina Rácz, Pál Salamon

**Affiliations:** 1 Faculty of Natural Sciences, University of Pécs, Ifjúság Útja 6, 7624 Pécs, Hungary; 2 Doctoral School of Health Sciences, University of Pécs, Vörösmarty M. Str. 4, 7621 Pécs, Hungary; 3 Department of Bioengineering, Faculty of Economics, Socio-Human Sciences and Engineering, Sapientia Hungarian University of Transylvania, 1 Libertatii Sq, 530104 Miercurea Ciuc, Romania

**Keywords:** adolescents, sport participation, psychological well-being, depressive symptoms, socioeconomic status

## Abstract

**Highlights:**

**What are the main findings?**

- Adolescent boys participating in organized sports demonstrated higher academic performance and greater life satisfaction compared to non-athletes, alongside more favorable socioeconomic family backgrounds.
- At the same time, athletes (particularly those engaged in competitive sports) reported slightly higher depressive symptom levels and more frequent sleep disturbances, indicating concurrent psychological strain.

**What is the implication of the main finding?**

- Organized sports during adolescence should be viewed as a dual-context environment, providing developmental benefits while also carrying potential psychological risks.
- Educational and youth sport programs may benefit from integrating mental health awareness and supportive coaching strategies, especially for competitively active and socially vulnerable students.

**Abstract:**

Background: Adolescence is a sensitive period for psychological, academic, and social development, and sports participation has been described as a potential protective factor for academic performance and psychological well-being. However, limited research has examined the combined influence of sports involvement, sport type, and family background on adolescents’ academic and psychological outcomes. This study aimed to investigate the associations between organized sport participation, sport type (football vs. judo), psychological well-being, psychosomatic symptoms, academic performance, and family socioeconomic background among adolescent boys. Methods: The sample consisted of 52 boys aged 11–14 years from a rural school, divided into football players (*n* = 13), judo athletes (*n* = 13), non-athletes (*n* = 13), and a contextual subgroup of students with special educational needs (SEN; *n* = 13), with the latter included for exploratory purposes only. Data included school-record-based academic performance and validated self-report measures of life satisfaction, depressive symptoms, psychosomatic complaints, perceived physical fitness, and socioeconomic background. Results: Athletes demonstrated significantly higher academic achievement than non-athletes in overall grade point average (*p* < 0.001), mathematics (*p* < 0.001), Romanian (*p* < 0.001), English (*p* = 0.03), and Hungarian (*p* < 0.001). They also reported higher life satisfaction (*p* < 0.001) but simultaneously showed slightly elevated depressive symptom scores (*p* < 0.001), indicating a paradoxical pattern of concurrent psychosocial benefits and psychological strain. Parental education (*p* < 0.001), parental occupational status (*p* = 0.01), and fathers’ occupational position (*p* = 0.02) were significantly higher among athletes’ families. Perceived physical fitness was also rated higher by athletes (*p* < 0.001). No significant differences were found in body mass index, family structure, or most psychosomatic symptoms. Conclusions: Sport participation was associated with more favorable academic and psychological indicators, yet also with elevated depressive symptoms, highlighting the dual nature of organized sport during adolescence. Future research should apply longitudinal designs, include female participants, and incorporate objective indicators of training load.

## 1. Introduction

Physical activity and socio-economic background are key determinants of adolescents’ mental health and development, particularly parental educational attainment and the broader family environment. Previous research has shown that the parents of student athletes tend to have higher academic and occupational status, which is associated with greater life satisfaction and better psychological well-being among their children [1]. Family background, including parental education and occupation, therefore represents a potential moderating factor in the associations between sport participation, mental health, and academic performance [2,3]. Psychological well-being during adolescence has been widely studied in both athletes and non-athletes, with evidence suggesting that sport participation may be associated with higher life satisfaction but also with increased psychological demands, particularly in competitive settings.

Regular physical activity contributes fundamentally to children’s physical and psychological development. It is defined as any bodily movement produced by skeletal muscles that results in energy expenditure and includes a wide range of behaviors such as active play, transportation, household activities, physical education, and organized sport participation [4,5]. According to the World Health Organization (WHO), physical activity supports overall health by preventing chronic diseases, promoting cardiovascular and metabolic functioning, and enhancing memory and cognitive performance [6,7,8]. It also provides psychological benefits, including reduced anxiety and depressive symptoms and improved mood [9]. Current public health guidelines recommend at least 150 min of moderate-intensity or 75 min of vigorous-intensity aerobic physical activity per week for youth [6]. Throughout this study, a clear distinction is made between general physical activity and organized sport participation, the latter referring specifically to structured, competitive, and socially embedded forms of activity.

Sport, as a structured form of physical activity, supports not only the development of physical abilities but also the strengthening of social competencies and mental health. Numerous studies have demonstrated that sports participation enhances physical fitness, social functioning, and psychological well-being during childhood and adolescence [10,11]. Engagement in sport can increase self-esteem, improve emotional regulation, and foster cooperation skills, thereby contributing to overall well-being [12,13].

Different types of sport may exert distinct effects on mental health. Some studies suggest that team sports are associated with lower perceived stress, reduced anxiety, higher motivation, and stronger social support, all of which may protect against depressive symptoms [14]. In contrast, individual sports, while promoting concentration, responsibility, and independence, often involve higher performance expectations that may trigger feelings of guilt or shame following failure [15]. In judo, for example, athletes frequently experience increased psychological strain during competitive situations and in the aftermath of losses, which may elevate the risk of depressive symptoms [16]. In football, athletes are exposed to continuous performance evaluation within a team context, where individual mistakes are publicly visible and may affect collective outcomes, generating social pressure and emotional stress, particularly in competitive environments [17]. Football and judo were selected to represent two contrasting sport contexts: football as a team-based discipline characterized by social embedding and shared responsibility, and judo as an individual combat sport associated with heightened personal accountability and performance pressure.

Although regular sport participation is linked to several psychological benefits, paradoxically, multiple studies have reported higher depressive symptom scores among athletes, particularly those engaged in competitive sports [16,18]. This phenomenon may be related to factors such as performance pressure, high training and competition loads, the physical and psychological consequences of sports injuries, and uncertainties surrounding athletic careers. Collectively, these factors can influence both mental and physical well-being and may be reflected in the psychosomatic symptoms observed in athletic populations.

Among athletes, regular physical exertion generally supports good physical condition; however, specific psychosomatic complaints such as sleep disturbances or fatigue may occur more frequently due to high training and competition loads [19,20]. The present study was conducted among Hungarian-speaking adolescents attending a rural lower secondary school in Romania. Previous educational research suggests that the academic trajectories and future orientations of Hungarian minority students are shaped by the combined effects of rural residence, family socioeconomic background, and institutional opportunities. These contextual factors may also influence access to organized sport and patterns of psychosocial development. Consequently, this population remains underrepresented in research on adolescent sport participation and mental well-being, and the psychosocial role of organized sport in this context has received limited empirical attention [21].

Our study also included students with mild learning difficulties who are not exempt from physical education. These students were included for contextual and exploratory purposes only and are not intended to constitute a fully comparable analytical group. Placement in this category may be initiated by parents or teachers, with the final decision made by a specialist committee that evaluates individual needs and developmental potential. Although these students participate in physical education classes, they typically engage in lower levels of physical activity and differ from both athlete and non-athlete peers on several indicators, which justifies examining them as a separate subgroup [22].

In Romania, students with special educational needs (SEN) are those who, due to persistent or temporary difficulties, require exceptional pedagogical support as determined by a multidisciplinary expert committee. The SEN category primarily includes learning disorders, as well as mild intellectual, motor, or sensory difficulties, along with speech, behavioral, or emotional problems.

Significantly, the learning difficulties characteristic of SEN students naturally influence their academic performance. Therefore, in this study, their school achievement is not interpreted as a direct basis for comparison but rather as contextual information. The present study addresses a research gap by examining sport participation, sport type, socioeconomic background, and perceived physical fitness jointly in a rural minority adolescent sample. We aimed to explore their psychological well-being, life satisfaction, psychosomatic symptoms, and socioeconomic background and to examine how these factors relate to different forms of sport participation.

As the joint examination of sport participation, sport type, and family background in rural minority contexts has so far been limited, several important research gaps can be identified in this field. First, although previous studies have reported differences in psychological well-being between athletes and non-athletes, little is known about how these patterns manifest among Hungarian-speaking adolescents living in rural areas of Romania. Second, the associations between team versus individual sports and psychological well-being or psychosomatic symptoms remain underexplored in this population. Third, the combined role of perceived physical fitness and family socioeconomic background in shaping these relationships has rarely been examined. Finally, the psychosocial profile of students with special educational needs has received limited empirical attention in sport-related research. The study focuses exclusively on boys because organized sport participation among girls in the investigated rural setting was extremely low, which would have precluded meaningful subgroup comparisons.

### Research Questions

1. Do adolescents participating in different sports differ from non-athletes in terms of psychological well-being (life satisfaction, depressive symptoms, psychosomatic complaints)?
2. Is there an association between sport participation (both presence and type) and students’ socioeconomic background (parental education, occupation, financial status)?
3. Do perceived physical fitness levels differ between athletes and non-athletes?
4. How can the outcomes of students with special educational needs (SEN) be contextualized in relation to athletes and non-athletes?

## 2. Materials and Methods

### 2.1. Study Design

The study was conducted at a rural lower secondary school in Sândominic, Harghita County, situated in the Eastern Carpathians region of Romania. Data were collected between September 2022 and July 2024. This observational, school-based study examined the relationships among sport participation, academic performance, and psychosocial well-being in early adolescence. The study followed a cross-sectional design and did not include any interventional component. The entire school population (*n* = 200; 103 females and 97 males) was initially screened using standardized questionnaires assessing the frequency, type, and context of physical activity and sport participation. During preliminary data screening, girls were excluded from the final sample because only a small proportion participated in regular, organized sports. Of the 97 eligible boys, 45 were excluded due to incomplete questionnaires, absence during data collection, or missing parental consent, resulting in a final sample of 52 participants. This substantial imbalance would have prevented meaningful statistical comparisons between sport-active and sport-inactive groups. Furthermore, sex-related biological and psychosocial differences, such as variations in pubertal development, motivation for physical activity, and social influences, could introduce additional confounding factors. For these reasons, limiting the sample to boys ensured greater group homogeneity and enabled clearer, more reliable interpretation of the associations among sport participation, academic performance, and psychological well-being.

### 2.2. Participants

The final sample comprised 52 male adolescents aged 11–14 years (mean ± SD: 13.5 ± 0.5 years). Participants were categorized into four subgroups based on sport participation status and educational characteristics: football players (*n* = 13), judo athletes (*n* = 13), students with special educational needs (SEN; *n* = 13), and a non-athlete control group (*n* = 13).

Football players were members of local clubs, training three to four times per week for approximately 90 min per session and participating in two official matches per week during the active season. Judo athletes trained within the judo division of a sports school, attending three 90 min sessions per week, and competing in approximately two to three official tournaments per month. Football and judo were selected to represent two distinct sport environments, as these were the most commonly practiced organized sports in the rural setting under investigation. Football, as a team sport, reflects a context characterized by shared responsibility, continuous social interaction, and collective performance goals. In contrast, judo, as an individual combat sport, involves heightened personal accountability, direct performance evaluation, and substantial competitive pressure. This contrast enabled the examination of sport-type-specific psychosocial patterns within an identical developmental and sociocultural context. The non-athlete control group and the SEN subgroup did not participate in organized sports; their physical activity consisted of regular physical education classes and daily household activities. The inclusion of SEN students enabled a broader pedagogical interpretation of the relationships between sport participation, psychological well-being, and academic performance within an inclusive rural minority educational context. Although their results did not provide a fully comparable analytical basis, their presence contributed to an interpretative framework that extended beyond patterns observed in typically developing students. Individuals in the SEN subgroup were formally recognized as having special educational needs under Romanian Ministry of Education regulations following multidisciplinary evaluation and official certification. The study was approved by the ethics committee of the Sapientia Hungarian University of Transylvania (approval no. 1/2024). Written informed consent was obtained from all participants and their legal guardians, in accordance with the Declaration of Helsinki.

### 2.3. Measures

This study used validated tools and standard methods to assess both objective and subjective indicators of academic performance, mental well-being, physical health, and socioeconomic status. All checks were conducted in accordance with established reliability and validity standards.

#### 2.3.1. Anthropometric Measurements

Height and body weight were measured following a standardized protocol, with participants barefoot and wearing light clothing. Body weight was measured using a digital scale (Omron BF214; Omron Healthcare, Kyoto, Japan), and height was assessed with a portable stadiometer (Seca 213; Seca GmbH & Co. KG., Hamburg, Germany). Body mass index (BMI) was calculated as weight (kg)/[height (m)]^2^. BMI was classified using WHO age-adjusted percentiles [6]: underweight (<5th percentile), healthy weight (5th–84th percentile), overweight (85th–94th percentile), and obesity (≥95th percentile). BMI is a widely used, non-invasive indicator of body composition and overall health status (CDC, 2024). Although BMI provides a general indicator of body composition, it does not directly reflect sport participation or fitness-related physiological adaptations. No additional performance-based or training-load measures (e.g., endurance capacity, strength tests, weekly training volume) were collected; therefore, anthropometric variables are interpreted as background health indicators rather than as physiological measures of sport participation.

#### 2.3.2. Academic Performance

Participants’ academic results were obtained from official school records to ensure objective and accurate measurement. For the analyses, the overall grade point average was used, along with subject-specific averages in mathematics, Romanian language, English language, and Hungarian language. It is important to note that academic performance was assessed solely on school grades rather than standardized tests. As grades rely on teacher evaluation, they inherently involve a degree of subjectivity and may vary across educational contexts, potentially introducing bias. This subjectivity may reduce the internal validity of academic performance measurement and partly account for the group differences observed in the analyses. Accordingly, findings on academic achievement should be interpreted with greater caution.

#### 2.3.3. Sociodemographic Data

Sociodemographic characteristics were assessed using a validated questionnaire adapted for adolescent populations, previously employed in the Health Behaviour in School-aged Children (HBSC) international research program [23]. The questionnaire measured the following variables: age, family structure (with whom the student resides), parents’ highest educational attainment, parental occupational status, and perceived family financial status. Perceived financial status was rated on a 5-point Likert scale (1 = very poor, 5 = very good).

#### 2.3.4. Health Status and Psychosomatic Symptoms

Health status was assessed using the Hungarian adaptation of the Adolescent Health Questionnaire validated by Pikó and Keresztes (2006) [19]. This instrument evaluates participants’ current health status, self-perceived physical fitness, and the frequency of psychosomatic symptoms such as headaches, back pain, sleep disturbances, fatigue, stomach complaints, and palpitations experienced in recent months.

#### 2.3.5. Psychological Variables

Life satisfaction was measured using an adapted version of the widely applied and validated Satisfaction with Life Scale (SWLS) developed by Diener et al. (1985) [24]. The SWLS consists of 5 items that assess overall life satisfaction. Responses were given on a 7-point Likert scale (1 = strongly disagree, 7 = strongly agree), with higher scores indicating greater life satisfaction.

Depressive symptoms were assessed using validated items incorporated into the questionnaire by Pikó and Keresztes (2006) [19]. The CES-D scale range (0–60) and interpretation of higher scores as indicators of more severe symptomatology were added. These items assess the frequency of symptoms such as sadness, apathy, difficulty concentrating, and decision-making difficulties. Responses were recorded on a 5-point Likert scale. As the instrument is intended as a screening tool rather than for clinical diagnosis, the obtained scores were interpreted as reflecting subclinical variability in depressive symptoms.

### 2.4. Data Collection Procedure

Data collection took place in classroom settings during regular school hours. The questionnaire battery required approximately 30 min to complete and was administered in a single session in paper-and-pencil format. Students with special educational needs (SEN) received additional support to ensure they understood all items.

The questionnaires were administered face-to-face under the immediate supervision of the research team to support accurate understanding and completion. All data-collection conditions were standardized across participants to minimize potential sources of bias. Composite variables were derived from questionnaire responses to ensure consistent processing of psychosocial characteristics. Data quality, validity, and reliability were verified using the statistical procedures described in Section 3 (Results).

### 2.5. Statistical Analysis

All data processing and statistical analyses were conducted using R version 4.4.1. Before hypothesis testing, data were cleaned and checked for accuracy, and distributional assumptions were evaluated using visual inspection (histograms, Q–Q plots) and the Shapiro–Wilk test. Because most variables violated normality assumptions, nonparametric statistical methods were used.

Group differences between athletes and non-athletes were examined using the Mann–Whitney U test. Comparisons among the four subgroups (control, SEN, football, and judo) were performed using the Kruskal–Wallis test, followed by pairwise Wilcoxon rank-sum tests where appropriate. Descriptive statistics are presented as medians and interquartile ranges. Group differences were visualized using boxplots generated with the ggpubr package.

All exact *p*-values are reported. As the study is exploratory and the sample size is limited, no correction for multiple comparisons was applied, and effect sizes were not calculated; these decisions are acknowledged as methodological limitations. The level of statistical significance was set at α = 0.05.

## 3. Results

### 3.1. Comparison Between Athletes and Non-Athletes

Significant differences were observed between athletes and non-athletes across several academic, socioeconomic, and psychological variables (Table 1).

Significant differences were observed between athletes and non-athletes across several academic, socioeconomic, physical, and psychological indicators (Table 1). Overall, athletes showed higher median academic grades in all assessed school subjects (*p* < 0.001–0.03). Life satisfaction scores were also significantly higher among athletes (*p* < 0.001), whereas their depressive symptom scores, although still within a non-clinical range, were slightly elevated compared to non-athletes (*p* < 0.001).

Regarding socioeconomic variables, parental educational attainment and parental occupational status were significantly higher in the athlete group (*p* < 0.001–0.02). Athletes also evaluated their physical fitness more positively (*p* < 0.001) and reported fewer school absences due to illness (*p* = 0.01).

### 3.2. Non-Significant Outcome Measures in Athletes and Non-Athletes

Non-significant differences were also examined to provide a complete overview of the measured variables. No statistically significant differences were found between athletes and non-athletes regarding body mass index (BMI) (*p* = 0.13), family structure (*p* = 0.34), or mothers’ occupational status (*p* = 0.10). Similarly, most psychosomatic complaints such as headaches (*p* = 0.78), diarrhea due to nervousness (*p* = 1.00), rapid heartbeat (*p* = 0.29), heartburn or stomach pain (*p* = 0.41), back pain (*p* = 0.05), sleep disturbances (*p* = 0.10), and fatigue (*p* = 0.08) did not differ significantly between the two groups.

Although these findings were not statistically significant, the borderline *p*-values for back pain, sleep disturbances, and fatigue should be interpreted cautiously and considered preliminary indications requiring confirmation in larger samples. These non-significant findings are reported in Table 2 to ensure transparency and completeness of the results.

### 3.3. Results of the Comparison Between the Four Subgroups (Control, Students with Special Educational Needs, Football Players, Judo Athletes)

For further analysis, participants were divided into four subgroups: the athlete group was split into judo athletes and football players, while the non-athlete group comprised regular students and those with special educational needs (SEN). This classification allowed for a more detailed examination of how sport type and differences in educational needs influence academic performance, psychological well-being, and socio-demographic background.

The results concerning students with special educational needs (SEN) should be interpreted with caution. The inclusion of this subgroup was not intended for direct statistical comparison but served contextual and exploratory purposes only. Although the SEN group consisted solely of boys, it included students with different types and degrees of learning difficulties or support needs. Given the small subgroup size and heterogeneity of support needs, SEN-related results are presented descriptively and should be interpreted cautiously.

**Figure 1:**
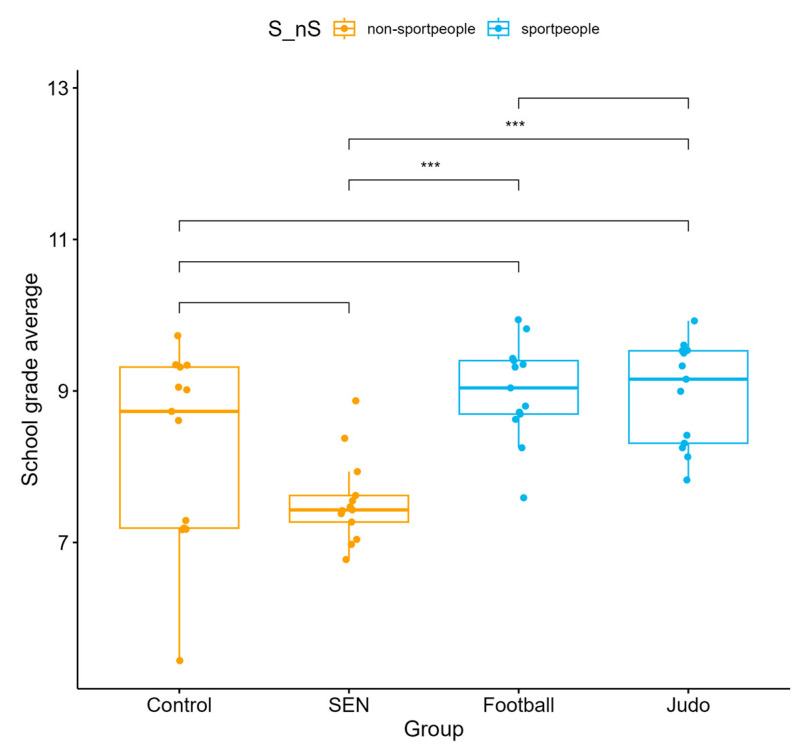
Annual academic grade point average across four subgroups: control, special educational needs, football, and judo (*** *p* < 0.001).

Significant differences were observed between the four subgroups in annual academic grade point average (*p* < 0.001). Football players (median = 9.04; IQR: 8.695–9.40) and judo athletes (median = 9.155; IQR: 8.31–9.53) consistently achieved higher scores than the control and SEN groups. SEN students had the lowest median values, while the control group fell within the intermediate range (Figure 1).

**Figure 2:**
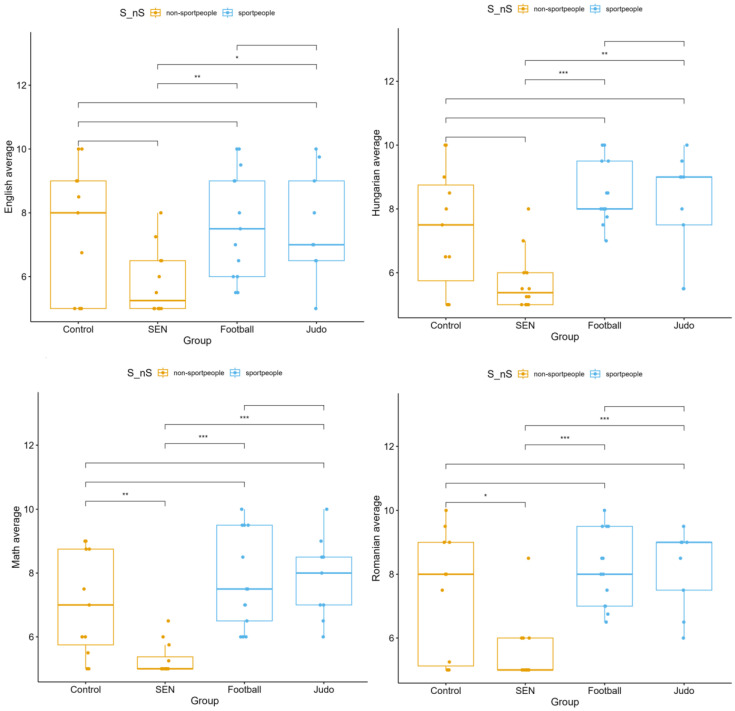
Subject-specific academic performance (Mathematics, Romanian, Hungarian, English) across four subgroups: control, students with special educational needs (SEN), football, and judo (* *p* < 0.05, ** *p* < 0.01, *** *p* < 0.001).

For all subjects assessed, significant differences were found between the four subgroups: mathematics (*p* < 0.001), Romanian language (*p* = 0.001), English language (*p* = 0.028), and Hungarian language (*p* = 0.001). Football players and judo athletes outperformed the control and SEN groups in each subject, with SEN students showing the lowest scores, particularly in mathematics and Hungarian language. The control group’s performance was generally in the mid-range but surpassed that of SEN students in multiple subjects (Figure 2).

**Figure 3:**
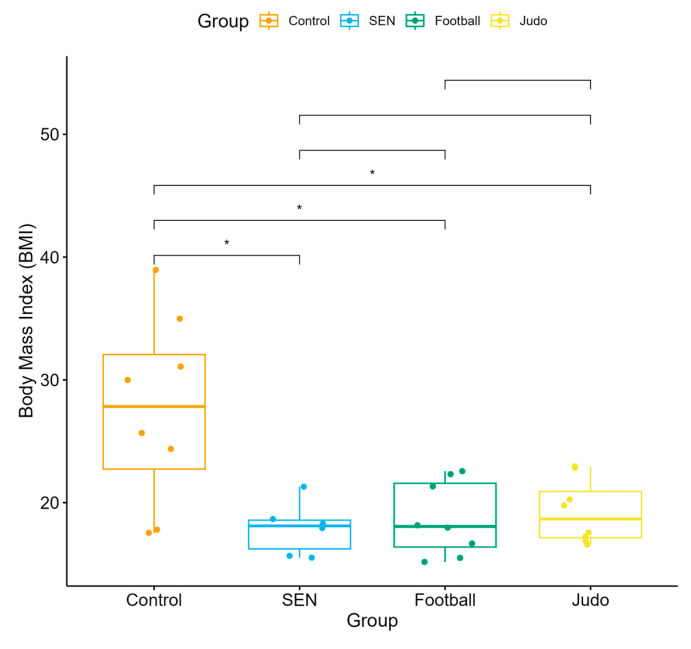
Body Mass Index (BMI) distribution across four subgroups: control, special educational needs, football, and judo groups (* *p* < 0.05).

BMI values were interpreted using WHO age-adjusted percentiles. The median BMI of the control group (27.83; IQR: 22.735–32.065) fell within the upper age-adjusted percentile range according to WHO criteria, whereas the athletic subgroups (football: 18.06; IQR: 16.385–21.58; judo: 18.67; IQR: 17.143–20.915) and the SEN group (18.11; IQR: 16.235–18.575) were generally within the normal age-adjusted percentile range (Figure 3).

**Figure 4:**
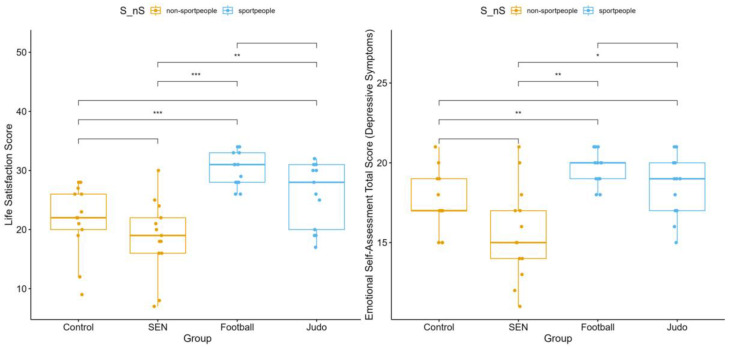
Life satisfaction and depressive symptoms were assessed in four subgroups: control, special educational needs, football, and judo groups (* *p* < 0.05, ** *p* < 0.01, *** *p* < 0.001).

Life satisfaction differed significantly between the groups (*p* < 0.001). Football players (median = 31; IQR: 28–33) and judo athletes (median = 28; IQR: 20–31) reported higher scores than the control group (median = 22; IQR: 20–26) and the SEN group (median = 19; IQR: 16–22). Depressive symptoms also differed (*p* < 0.001), with athletes scoring slightly higher than the non-athlete groups (Figure 4). These differences likely reflect subclinical variation and should not be interpreted as indicating clinical depression.

**Figure 5:**
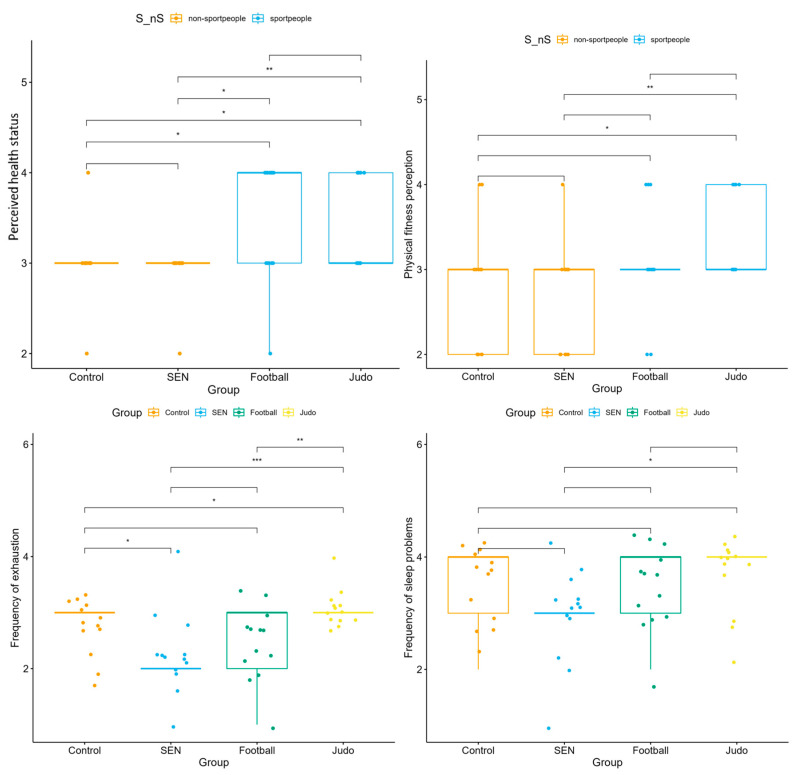
Health and Psychosomatic Symptoms were divided into four subgroups: control, special educational needs, football, and judo groups (* *p* < 0.05, ** *p* < 0.01, *** *p* < 0.001).

Perceived health status showed significant differences between the groups (*p* = 0.006) (Figure 5). The highest scores were recorded among football players (median = 4, IQR = 3–4), whereas the SEN and control groups reported lower values (median = 3, IQR = 3–3).

Self-rated physical fitness also differed (Figure 5) significantly (*p* = 0.024), with judo athletes (median = 3, IQR = 3–4) and football players (median = 3, IQR = 3–3) scoring higher than the SEN and control groups (median = 3, IQR = 2–3).

Sleep disturbances and fatigue differed significantly between groups, with the highest values observed in judo and football players, suggesting that intensive sport participation may be associated with increased psychological load (Figure 5).

**Figure 6:**
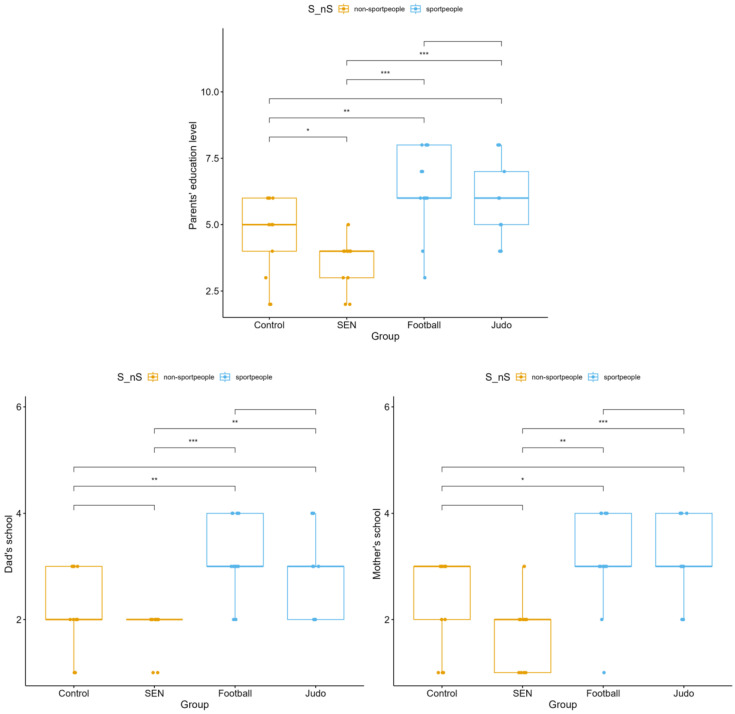
Parental (mothers and fathers) education levels are divided into four subgroups: control, special educational needs, football, and judo (* *p* < 0.05, ** *p* < 0.01, *** *p* < 0.001).

Significant differences were observed in the overall parental education level (*p* < 0.001), with higher scores reported in the sport-participating groups—particularly among football players and judo athletes—compared to the control and SEN groups (Figure 6). When examined separately, fathers’ (*p* < 0.001) and mothers’ (*p* = 0.001) education levels followed the same pattern, with both being higher in the athlete groups than in the non-athlete subgroups.

**Figure 7:**
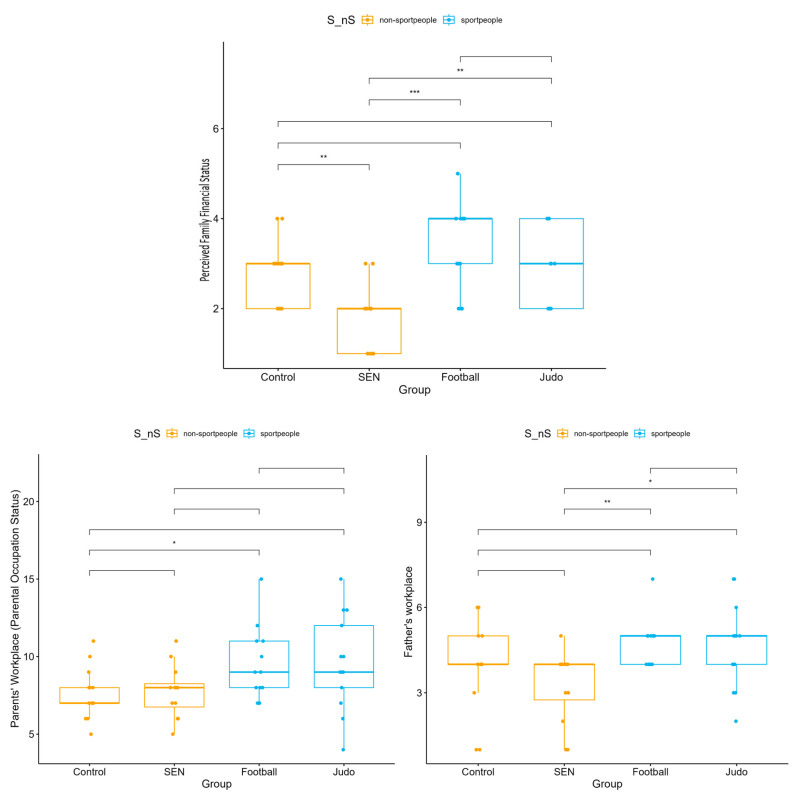
Parental occupation and perceived family financial status, four subgroups: control, special educational needs, football, and judo (* *p* < 0.05, ** *p* < 0.01, *** *p* < 0.001).

The father’s occupational position showed a significant difference (*p* = 0.032), with parents in the athlete groups holding higher-level positions (Figure 7). Perceived family financial status also differed significantly (*p* = 0.001), with the families of athlete students reporting more favorable circumstances than those in the control and SEN groups.

### 3.4. Non-Significant Outcome Measures Across the Four Subgroups (Control, SEN, Football, Judo)

No statistically significant differences were found between the groups in the occurrence of headache due to nervousness (*p* = 0.423), back pain (*p* = 0.285), heartburn or abdominal pain (*p* = 0.265), diarrhea due to nervousness (*p* = 0.716), or rapid heartbeat (*p* = 0.099). Similarly, no significant differences were observed in family structure (*p* = 0.712), parental occupational status (*p* = 0.063), or mother’s workplace (*p* = 0.175). These non-significant findings confirm that most psychosomatic complaints and family structure variables did not systematically vary across subgroups in this sample (Table 3).

## 4. Discussion

### 4.1. Discussion of the Main Results

The present study aimed to provide a comprehensive overview of the associations between sport participation, psychological well-being, psychosomatic symptoms, physical health, and socioeconomic background among adolescent boys. Comparing athletes, non-athletes, and students involved in different sports enabled interpretation of the role of sport participation across multiple dimensions, from academic performance to subjective well-being. The uniqueness of this investigation lies in its integrated approach, simultaneously examining the presence and type of sport participation, psychological indicators, and socioeconomic factors, while also contextualizing the situation of students with special educational needs.

The study’s findings are interpreted across two complementary analytical perspectives. At the group-comparison level, we examine general differences between athletes and non-athletes to identify overarching patterns related to sport participation. At the subgroup-comparison level, we analyze the four study groups (control, SEN, football, judo) to explore how sport type and educational status are associated with psychosocial and socioeconomic characteristics.

This dual approach ensures that broad trends become visible while also revealing subgroup-specific nuances that would otherwise go undetected with a single-level analytical strategy.

#### 4.1.1. Research Question I

Does the psychological well-being of adolescents differ between athletes and non-athletes, as well as across different types of sports (life satisfaction, depressive symptoms, psychosomatic complaints)?

At the macro level, significant differences were observed between athletes and non-athletes in both life satisfaction and depressive symptoms. The median life satisfaction score was significantly higher among athletes compared to their non-athletic peers. At the same time, depressive symptom scores were also higher in the athlete group than in the non-athlete group. This dual pattern, where higher life satisfaction coexists with higher depressive symptom scores, aligns with the paradox described in the international literature, which suggests that while sports participation offers numerous psychological benefits, it can also be associated with increased psychological strain [17].

The micro-level analysis, which compared the four subgroups, provided a more nuanced perspective. The highest life satisfaction scores were observed among football players and judo athletes, while the lowest scores were recorded in the SEN group. The control group’s median score fell between these two extremes. Regarding depressive symptoms, the highest scores were found among football players, followed by judo athletes, whereas the lowest scores were reported in the SEN group.

As the instrument applied is designed as a screening tool rather than for clinical diagnosis, the results are interpreted not as depression but as indicators of subclinical variability in emotional strain. The co-occurrence of positive well-being indicators and elevated emotional symptoms has also been documented in previous studies among adolescent athlete populations. This is consistent with the results of Eather et al. (2023) [25], who found that teenage athletes exhibited both higher psychological well-being and greater psychological distress compared to their non-athlete peers. Previous studies suggest that competitive sport environments may involve psychological demands related to performance expectations, perceived failure, and injury experiences, which can contribute to increased emotional strain in adolescent athletes [26,27,28,29].

Recent findings have also shown that the prevalence of depressive symptoms varies by sport type. Multiple studies have demonstrated that athletes competing in individual sports are more prone to depressive symptoms compared to those in team sports [30]. From a psychological perspective, several factors may explain why athletes in individual sports could be at greater risk for depression. One key aspect is the difference in how success and failure are attributed. Hanrahan and Cerin (2009) found that individual and team sport athletes exhibit distinct attributional styles [31]. Specifically, athletes in individual sports tend to engage more in internal attribution.

At the macro level, athletes rated their health status and physical fitness significantly more favorably than non-athletes, while no substantial differences were observed for other psychosomatic symptoms. At the micro level, football players reported the highest scores for both health status and physical fitness, whereas the lowest scores were found in the control and SEN groups. A significant difference emerged in the frequency of sleep problems and fatigue: the former occurred most frequently among football players, whereas fatigue was least prevalent in the SEN group. These findings indicate that athlete status is generally associated with more favorable self-assessments of health and fitness; however, competitive sport is concurrently related to small but significant differences in selected psychosomatic symptoms, particularly sleep problems and fatigue. This is consistent with previous research indicating that regular physical activity enhances subjective health status and physical fitness [15]. However, several studies have shown that sleep problems and fatigue are common among athletes. For example, Doherty et al. (2021) reported that sleep disturbances and fatigue may occur among adolescent athletes, particularly during periods of elevated training load [32]. Overall, the findings addressing this research question indicate that sports participation positively influences life satisfaction; however, engagement in competitive sports may also be associated with higher emotional strain. The type of sport entails distinct psychological risks and protective factors. At the same time, the outcomes observed in students with special educational needs (SEN) provide important contextual insights, demonstrating that patterns of psychological well-being can vary in the presence of specific learning requirements. This complex pattern aligns with the literature’s conceptual framework, which posits that sports participation should not be considered a protective factor in isolation; rather, it represents a multidimensional environment that simultaneously supports and challenges adolescents’ psychological functioning [17].

#### 4.1.2. Research Question II

Is students’ sports participation status and choice of sport associated with their family’s socioeconomic background (parental education, occupation, financial situation) and their academic performance?

The study’s findings indicate that sports participation is not merely a matter of individual choice but is closely linked to opportunities and resources derived from family background [33]. At the macro level, the parents of student athletes demonstrated higher educational attainment, a more favorable occupational status, and better financial conditions than the parents of non-athlete peers. This pattern is consistent with international evidence indicating that higher socioeconomic status families can access sports participation more easily, both through financial investment (equipment, travel, membership fees) and logistical support [34].

At the micro level, differences between sports disciplines revealed even more pronounced social patterns. Both football and judo athletes came from families with more favorable socioeconomic indicators compared to the control group and, in particular, the SEN group. Although football is generally considered a more socially accessible sport, regular competition, equipment requirements, and the travel associated with training still demand significant resources in practice. In judo, the club structure, training organization, and participation in competitions traditionally entail higher financial and logistical burdens, which helps explain the higher socioeconomic status observed among athletes’ families [35].

The socioeconomic background of students with special educational needs (SEN) consistently presented the most disadvantaged profile. This not only explains the lower rates of sports participation among this group but also highlights that access to sports infrastructure is organized along lines of social inequality. Therefore, the lower sports participation observed among SEN students cannot be attributed solely to pedagogical or psychological factors; it also reflects underlying socioeconomic disparities [36]. This is further supported by consistent empirical evidence indicating that low socioeconomic status is one of the strongest negative predictors of sports participation in childhood and adolescence, partly due to the lack of financial, infrastructural, and parental support resources [37].

Overall, the findings suggest that both the presence and type of sports participation are closely embedded within the social context of families. The more favorable background characteristics observed among athletes are not merely statistical differences but reflect structural relationships in which sport can function as a conduit for the intergenerational transmission of social advantages. These results align with research that conceptualizes sports participation as an indicator of social resources and emphasizes that access to organized sports activities is unevenly distributed among youth [38]. These differences in social and familial background are also reflected in academic performance. The association between students’ sports participation and their educational outcomes highlights that organized sports engagement contributes not only through physical or psychological mechanisms but is also closely linked to the family’s socioeconomic status. Student-athletes from higher socioeconomic backgrounds demonstrated superior academic performance, consistent with previous research emphasizing the relationship between socioeconomic advantages and school achievement [39]. The exceptional academic performance of athletes from higher socioeconomic backgrounds is consistent with previous research emphasizing the relationship between socioeconomic advantages and school achievement [40].

#### 4.1.3. Research Question III

Does perceived physical fitness differ between athletes and non-athletes, as well as across different sports disciplines?

The results of the study clearly indicated that athlete status is significantly associated with adolescents’ perceived physical fitness, with athletes reporting substantially higher self-assessments of their physical condition compared to non-athlete students. This difference aligns well with international literature, which suggests that regular training, the development of motor competencies, and the positive experiences associated with sports participation favorably influence bodily self-efficacy and perceived fitness [35]. The more positive self-assessments observed among athletes likely stem not only from actual physical advantages but also from the identity associated with sports participation, social reinforcement, and regular performance feedback.

The micro-level comparison further nuanced this relationship: football players exhibited the highest perceived fitness, followed by judo athletes, while the lowest values were observed in the control and SEN groups. The exceptionally positive self-assessments of football players may be related to the complex physical demands of the sport (speed, endurance, and agility) as well as the communal experience inherent in team sports, which can further enhance the sense of bodily competence [41]. Similarly, the technical and physical characteristics of judo, such as body control, strength, and coordination demands, may contribute to a favorable perception of fitness among judo athletes. In contrast, the lower values observed among non-athlete and SEN students suggest that the absence of regular physical activity, combined with lower physical self-confidence, may jointly contribute to a less favorable perception of fitness.

#### 4.1.4. Research Question IV

How can the outcomes of students with special educational needs (SEN) be contextualized in relation to athletes and non-athletes?

The inclusion of the SEN group was contextual and exploratory, providing descriptive information on the psychosocial characteristics of adolescents with special educational needs. Given the limited empirical evidence in this field, the present findings primarily outline relevant educational and psychosocial patterns. The observed results suggest that social inequalities and learning difficulties may be accompanied by complex processes related to self-reporting and expectations, which merit further investigation using longitudinal and qualitative approaches. This group exhibited the most disadvantaged socio-economic background, reinforcing the observation that the presence of special educational needs is frequently associated with a lower socio-economic status. This disadvantage is also reflected in the lowest academic achievement, which aligns with their educational difficulties and highlights why access to sports infrastructure is organized along socio-economic inequalities [42].

Since SEN students often face lower external performance expectations due to their learning difficulties, this reduced external pressure may protect them from performance-induced anxiety and depression typically observed in competitive athletes. The differential setting of goals and expectations can thus be interpreted as a protective mechanism that mitigates psychological strain [43]. In examining mental health, we observed a complex pattern: SEN students reported low life satisfaction scores, yet their self-reported depressive symptom scores were also low. Two non-exclusive explanatory hypotheses may account for this discrepancy. First, methodological factors may play a role: due to learning difficulties, adolescents may find it more challenging to identify and verbalize their internal symptoms, potentially leading to underreporting bias. Second, a possible expectation-based protective mechanism may be operative, whereby SEN students face lower external performance demands, which likely reduces performance-induced psychological strain, such as anxiety or depressive symptoms [44]. Overall, the findings for the SEN group highlight that psychological well-being and academic performance result from complex mechanisms: social disadvantage, limitations in self-report measurement, and differential expectations calibration all play a role. These findings underscore the need for further longitudinal and qualitative research to explore the mental health and sports participation opportunities within this special population.

### 4.2. Theoretical Significance and Practical Implications of This Study

It helps fill the picture of the physical and psychosocial characteristics of adolescent athletes and non-athletes. Other research focused only on the positive effects of sports participation. Results from this study showed that, although athletes had normal body mass index values, high self-rated health ratings, and good perceived physical fitness and life satisfaction, depressive symptoms in this group were not negligible. That means that being an athlete does not fully protect one from psychological strain. In fact, certain circumstances can even increase the risk, such as competitions in which coaches and parents explicitly impose performance pressure. It corroborates previous studies that emphasize the importance of sport-related psychological problems, such as pre-competition anxiety, injury-related absence, and internal attributional style in the mental health of young athletes. Therefore, our results extend theoretical models of the relationship between sport and psychological well-being by emphasizing that, notwithstanding the advantages of physical activity, potential psychological risk factors should also be considered.

Practically speaking, this study has several valid messages. It provides further evidence that widening participation in sports, especially team sports, is associated with better health indicators and a more satisfying life. In addition, it underscores the deliberate support of young athletes’ mental health through lessons on stress management and a psychologically safe coaching environment, as well as ensuring both parents and coaches communicate realistic expectations.

The significance of the present results is further reinforced by the specific sociocultural context of the sample, consisting of ethnic Hungarian adolescents living in rural regions of Romania. This underrepresented minority population faces limited educational resources and distinct cultural integration challenges, which may shape both sport participation opportunities and psychosocial development.

These findings suggest that prevention and intervention programs may benefit from addressing not only physical performance but also mental health. The main takeaway is that sport participation is associated with both beneficial and potentially challenging psychological processes, which should be considered in youth sport practice.

### 4.3. Limitations of This Study

Limitations are to be noted in this study. First, the sample was relatively small, particularly in some subgroups; this may have reduced power and, hence, the reliability of between-group comparisons. Second, our study comprises males only; thus, caution should be taken about generalizing the findings to female athletes or any mixed-gender population. Third, because this is a cross-sectional design, no causal inferences can be made about the relationships among athlete status, psychological well-being, and psychosomatic symptoms. Finally, possible bias arising from a self-reported questionnaire, for example, positive social desirability or inaccurate recall, cannot be ruled out. In addition, the subgroup of students with special educational needs (SEN) was included for descriptive and exploratory purposes, to provide contextual insight into diverse educational experiences rather than for direct comparison with the athlete or control groups. Therefore, the results concerning this subgroup should be interpreted with caution.

Furthermore, as the sample consisted solely of boys, future research should include female participants to examine potential gender differences in these associations. In addition, no objective indicators of physical fitness or training load (e.g., endurance tests, weekly training volume, competition intensity) were available, which limits the interpretation of sport-related physiological burden. Furthermore, data collection was conducted in an educational institution operating in a rural minority-language context, which may limit the generalizability of the findings to other educational and cultural settings. Institutional characteristics and local pedagogical practices may have influenced both academic evaluation and psychosocial self-report indicators. Dividing the small total sample into four equal-sized subgroups (n = 13 each) substantially reduced statistical power and increased the likelihood of Type II errors.

## 5. Conclusions

This cross-sectional study examined the associations between organized sport participation and academic, psychosocial, and socioeconomic characteristics in a small sample of 11–14-year-old boys attending a rural, minority-language school in Romania. Within this specific educational and sociocultural context, structured participation in football or judo was associated with higher academic performance, greater life satisfaction, and more favorable family socioeconomic indicators compared with non-athlete peers.

A key finding of the study is the identification of a paradoxical pattern in which athletes reported both higher life satisfaction and slightly elevated depressive symptom scores. These results suggest that, in this particular population, competitive sport may function simultaneously as a supportive and a psychologically demanding environment. The inclusion of students with special educational needs provided additional contextual insight into educational disadvantage; however, their results were interpreted descriptively only due to methodological constraints.

Given the exploratory design, the small and equal sized subgroups, and the reliance on self-reported measures, the present findings cannot be generalized beyond the examined rural minority setting. Nevertheless, they highlight the potential relevance of integrating psychological support components such as stress management strategies and monitoring of training load into sport programs implemented in similar educational and sociocultural environments.

## Figures and Tables

**Table 1:** Variables with statistically significant differences between athletes and non-athletes.

Variable	Non-Athletes		Athletes		Mann–Whitney U-Test *p*-Values
	**Median**	**IQR**	**Median**	**IQR**	
School grade average	7.51	7.21–8.83	9.09	8.46–9.48	<0.001
Math average	5.5	5–6.75	7.5	6.62–8.87	<0.001
Romanian average	5.25	5–8	8.5	7.12–9	<0.001
English average	6	5–8	7.25	6.5–9	0.03
Hungarian average	6	5–7.75	8.25	7.81–9.37	<0.001
Parents’ education level	4	3.25–5	6	5–7.75	<0.001
Parents’ Workplace (Parental Occupation Status)	8	7–8	9	8–11	0.01
Life Satisfaction Score	21	18–24.75	29.5	26–31	<0.001
Emotional Self-Assessment Total Score (Depressive Symptoms)	17	15–18	19	18–20	<0.001
Dad’s school	2	2–2	3	2–4	<0.001
Mother’s school	2	1.25–3	3	3–4	<0.001
Father’s workplace	4	3–4	5	4–5	0.02
Social relations	2	2–3	3	2.25–4	<0.001
Perceived Family Financial Status	3	3–3	3.5	3–4	<0.001
Physical fitness perception	3	2–3	3	3–4	<0.001
School absence due to illness	3	2–3	3	3–4	0.01

**Table 2:** Variables with no statistically significant differences between athletes and non-athletes.

Variable	Non-Athletes		Athletes		Mann–Whitney U-Test *p*-Values
	Median	IQR	Median	IQR	
Body Mass Index (BMI)	19.98	17.83–28.91	18.06	16.83–21.58	0.13
Family structure (Who do you live with?)	5	5–5	5	5–5	0.34
Mother’s workplace	4	3–5	5	3–5.75	0.10
Frequency of headaches due to nervousness	3	3–4	3	3–4	0.78
Frequency of back pain	3	2–3	3	3–4	0.05
Frequency of sleep problems	3	3–4	4	3–4	0.10
Frequency of exhaustion	2.5	2–3	3	3–3	0.08
Frequency of heartburn/stomach pain	3	2.25–4	4	3–4	0.41
Frequency of diarrhea due to nervousness	4	3–4	4	3–4	1.00
Frequency of rapid heartbeat	4	3–4	4	4–4	0.29

**Table 3:** Variables with no statistically significant differences between the four subgroups: control, special educational needs, football, and judo groups.

Variable	Control		SEN		Football		Judo		Kruskal–Wallis H-Test *p*-Values
	Median	IQR	Median	IQR	Median	IQR	Median	IQR	
Family structure (living arrangement)	5	5–5	5	5–5	5	5–5	5	5–5–5	0.712
Parents’ Workplace (Parental Occupation Status)	7	7–8	8	6.75–8.25	9	8–11	9	8–12–12	0.063
Mother’s workplace	3	3–4	4	3–5.5	5	3–6	5	4–5–5	0.175
Frequency of headaches due to nervousness	3	3–4	3	3–4	3	3–3	4	3–4–4	0.423
Frequency of back pain	3	2–3	2	2–4	3	3–4	3	3–4–4	0.285
Frequency of heartburn/stomach pain	3	3–4	3	2–4	3	2–4	4	3–4–4	0.265
Frequency of diarrhea due to nervousness	4	3–4	4	3–4	4	3–4	4	4–4–4	0.716
Frequency of rapid heartbeat	4	3–4	3	3–4	4	3–4	4	4–4–4	0.099

## Data Availability

Data are contained within the article.
